# Xenophagy as a Strategy for *Mycobacterium leprae* Elimination during Type 1 or Type 2 Leprosy Reactions: A Systematic Review

**DOI:** 10.3390/pathogens12121455

**Published:** 2023-12-15

**Authors:** Débora Dantas Nucci Cerqueira, Ana Letícia Silva Pereira, Ana Elisa Coelho da Costa, Tarcísio Joaquim de Souza, Matheus Santos de Sousa Fernandes, Fabrício Oliveira Souto, Patrícia d’Emery Alves Santos

**Affiliations:** 1 Department of Immunology, Keizo Asami Institute-iLIKA, Federal University of Pernambuco-UFPE, Recife 50670-901, Pernambuco, Brazil; debora.nucci@ufpe.br (D.D.N.C.); leticia.spereira@ufpe.br (A.L.S.P.); ana.elisa@ufpe.br (A.E.C.d.C.); matheus.sfernandes@ufpe.br (M.S.d.S.F.); fabricio.souto@ufpe.br (F.O.S.); 2 Postgraduate Program in Biology Applied to Health-PPGBAS, Federal University of Pernambuco-UFPE, Recife 50670-901, Pernambuco, Brazil; 3 Life Sciences Center-NCV, Agreste Academic Center-CAA, Federal University of Pernambuco-UFPE, Caruaru 55014-900, Pernambuco, Brazil; tarcisio.joaquim@ufpe.br

**Keywords:** *Mycobacterium leprae*, leprosy reactions, autophagy, xenophagy

## Abstract

Background: *Mycobacterium leprae* is an intracellular bacillus that causes leprosy, a neglected disease that affects macrophages and Schwann cells. Leprosy reactions are acute inflammatory responses to mycobacterial antigens, classified as type1 (T1R), a predominant cellular immune response, or type2 (T2R), a humoral phenomenon, leading to a high number of bacilli in infected cells and nerve structures. Xenophagy is a type of selective autophagy that targets intracellular bacteria for lysosomal degradation; however, its immune mechanisms during leprosy reactions are still unclear. This review summarizes the relationship between the autophagic process and *M. leprae* elimination during leprosy reactions. Methods: Three databases, PubMed/Medline (n = 91), Scopus (n = 73), and ScienceDirect (n = 124), were searched. After applying the eligibility criteria, articles were selected for independent peer reviewers in August 2023. Results: From a total of 288 studies retrieved, eight were included. In multibacillary (MB) patients who progressed to T1R, xenophagy blockade and increased inflammasome activation were observed, with IL-1β secretion before the reactional episode occurrence. On the other hand, recent data actually observed increased IL-15 levels before the reaction began, as well as IFN-γ production and xenophagy induction. Conclusion: Our search results showed a dichotomy in the T1R development and their relationship with xenophagy. No T2R studies were found.

## 1. Introduction

*Mycobacterium leprae* is an intracellular acid-fast bacillus that causes leprosy, a disease that affects the peripheral nerves, skin, eyes, and respiratory tract [1]. Despite medical advancements, leprosy is still an important public health problem in Brazil and worldwide due to its severe consequences [2]. Factors that contribute to this include the stigma related to the disease, a lack of understanding and knowledge, failure in early detection, a sub-notification of cases, and bacterial resistance to dapsone and rifampicin [3].

The Ridley–Jopling classification (1966) is one of the most widely used systems to classify leprosy, which divides patients into five groups, according to their clinical and immunological status [4]. Tuberculoid leprosy (TT) is characterized by a robust cellular immune response against *M. leprae* antigenic determinants, the presence of a single lesion, well-developed granulomas, and negative or rare bacilli [5]. In contrast, lepromatous leprosy (LL) is characterized by a strong humoral response that does not prevent bacterial proliferation and tends to clinically manifest with skin lesions and high bacterial load [6]. Most patients present borderline phenotypes, which are immunologically unstable: borderline tuberculoid (BT), borderline borderline (BB), and borderline lepromatous (BL). Indeterminate (II) cases are considered to represent the initial stage of the disease. These cases eventually move toward one of the poles but progression can be halted with treatment [7]. However, in 1998, the World Health Organization’s Leprosy Expert Committee established a practical and easy classification for treatment: paucibacillary (PB) cases are those in which the cutaneous lesions number does not exceed five, including TT forms, while multibacillary (MB) cases present six or more skin lesions, including LL forms [7].

During the course of the disease, patients may experience exacerbated inflammatory responses known as leprosy reactions, which can be classified into two distinct types [8]. Type 1 reactions (also called reversal reaction/RR) are characterized by an exacerbation of the cellular immune response against *M. leprae* antigenic determinants, with CD4+ T lymphocytes and CD163+ macrophage infiltration, and tend to occur more frequently in patients presenting the PB clinical forms, but RR can also affect BB and BL patients. On the other hand, type 2 reactions (erythema nodosum leprosum/ENL) are an exacerbation of the humoral immune response with tissue deposition of immune complexes and neutrophil infiltration, which are more commonly observed in patients presenting the BL and LL forms [9,10]. These reactions are the primary cause of irreversible nerve damage and anatomical deformities related to leprosy and may arise spontaneously in up to 50% of patients before, during, and after treatment [8].

Autophagy (Greek: *autos* = self + *phaguein* = eating) is the process through which cellular components are degraded or recycled within the lysosome [11]. Xenophagy (Greek: *xenos* = strange + *phaguein* = eating) is a specific type of selective autophagy related to the identification and removal of intracellular bacteria [12], aiding in the activation of the host’s innate and adaptive immune system as a way to limit exacerbated inflammation and control infection [13].

When bacteria infect host cells, they can be recognized among others by Pattern Recognition Receptors (PRRs) and subsequently labeled by ubiquitin in the cytosol [14]. The ubiquitinated pathogen is then recognized by a group of adaptors featuring a ubiquitin-binding domain and a LC3-interacting region motif, such as p62/SQSTM1 (sequestosome 1), optineurin (OPTN), and NDP52 (nuclear domain 10 protein 52), which bind the ubiquitinated cargo to LC3 (microtubule-associated protein 1 light chain 3) on autophagosomes [14,15]. Then, the maturation of the autophagosome occurs through the autophagy-related protein complex (ATG): ATG12-ATG5-ATG16L1 and other components [16]. LC3 is the main indicator of autophagic activity [17]. During the autophagy process, the cytosolic form of LC3 (LC3-I) conjugates with phosphatidylethanolamine (PE) through the ATG3 to form the LC3- phosphatidylethanolamine conjugate (LC3-II), which is located in pre-autophagosomes and autophagosomes, making this protein an autophagosome marker [17]. Furthermore, the adaptors can also target bacteria-residing vacuoles or damaged vacuolar membranes in a ubiquitin-independent manner. In this scenario, the adaptors can respond to a wide variety of protein-, lipid- or sugar-base signals, which encompass galectin, complementing protein C3 and nucleotide-binding oligomerization domain (NOD) proteins [14].

Xenophagy has been shown to play an immunological role in *M. leprae* control [18]. Specifically, patients with the TT form of leprosy have been found to exhibit higher levels of xenophagy compared to LL-form patients [19]. Inhibition of macrophage xenophagy and a strong anti-inflammatory immune response could contribute to the bacillus persistence in LL patients [5]. On the other hand, MB patients who developed type 1 reactions showed a restoration of autophagic flux, leading to a limited form of this episode [19]. However, studies on the role of xenophagy in *M*. *leprae* elimination remain limited. In this systematic review, we will explore the relationship between the autophagic process and *M. leprae* elimination during type 1 and type 2 leprosy reactions.

## 2. Methods

The present systematic review followed the Preferred Reporting Items for Systematic Reviews and Meta-Analysis (PRISMA) guidelines.

### 2.1. Study Selection and Eligibility Criteria

Eligibility criteria were previously used to minimize the risk of bias. The inclusion and exclusion criteria followed the PICOS (Population/Intervention/Control/ Outcomes/Study) (Table 1). There were no restrictions on language or publication date. Articles that did not meet the following eligibility criteria were excluded: (a) studies that use only mice and rats from different species; (b) studies that do not have a control group or comparator; (c) studies in animal models and/or other organisms; as well as reviews, letters for editors, duplicates, and the presence of data used in different studies.

### 2.2. Information Sources and Literature Search Strategies

The search strategy was carried out during the period from March to April 2023. The databases used were PubMed (Medline), Scopus, and Embase. The search strategies used were: PubMed (Medline): ((((*Mycobacterium leprae*) OR (Leprosy)) OR (Hansen’s Disease)) OR (Hansen Disease)) AND ((((Autophagy) OR (Autophagy, Cellular)) OR (Cellular Autophagy)) OR (Xenophagy)). In the Scopus and Science Direct databases, the following search equation was used: ((((“*Mycobacterium leprae*”) OR (“Leprosy”)) OR (“Hansen’s Disease”)) OR (“Hansen Disease”)) AND ((((“Autophagy”) OR (“Autophagy, Cellular”)) OR (“Cellular Autophagy”)) OR (“Xenophagy”)).

### 2.3. Selection and Data Collection

**Figure 1:**
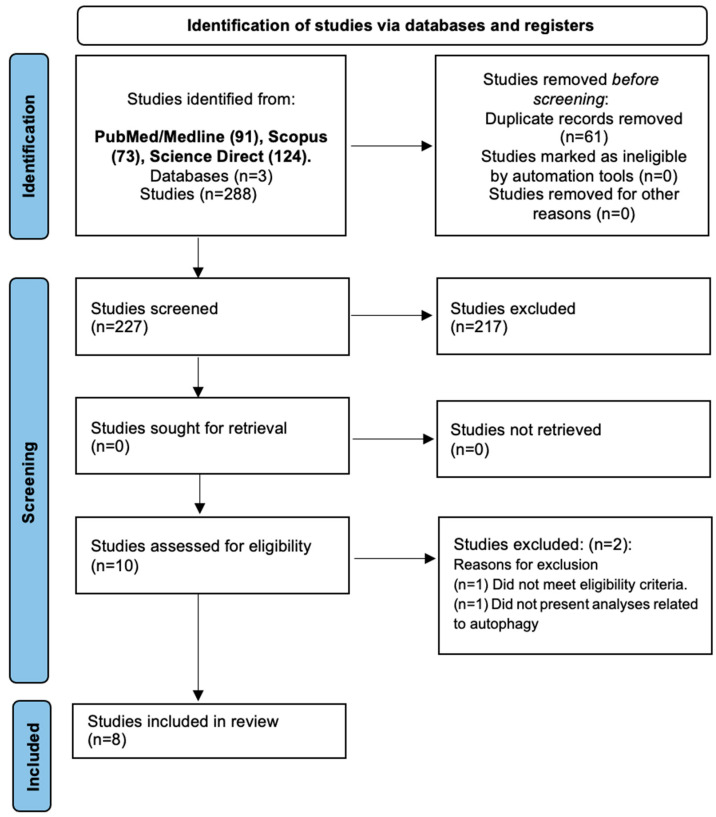
PRISMA 2020 flow diagram for new systematic reviews, which included searches of databases and registers only. We considered, if feasible, reporting the number of records identified from each database or register searched (rather than the total number across all databases/registers).

The screening of studies was performed through reading the titles, abstracts, and full texts. The selection of studies was performed by two independent researchers (D.D.N.C. and M.S.d.S.F.). Discrepancies were resolved by a third rater (P.d.A.S.) (Figure 1).

### 2.4. Data Items

Within the included articles, information related to authors, year of publication, study design, group, number of participants (n), sex, average age, average bacillary index (BI), and logarithmic bacillary index of skin lesion (LBI) was extracted. Furthermore, information about patients with leprosy and their treatment status was also extracted. Finally, information was obtained about the cell types used and the outcomes linked to autophagy/xenophagy in in vitro studies and in humans with leprosy.

### 2.5. Risk of Bias Assessment

**Figure 2:**
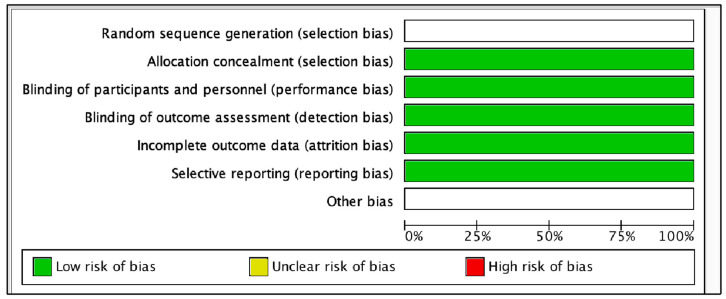
Risk of bias graph: a review of authors’ judgments about each risk of bias item presented as percentages across all included studies.

**Figure 3:**
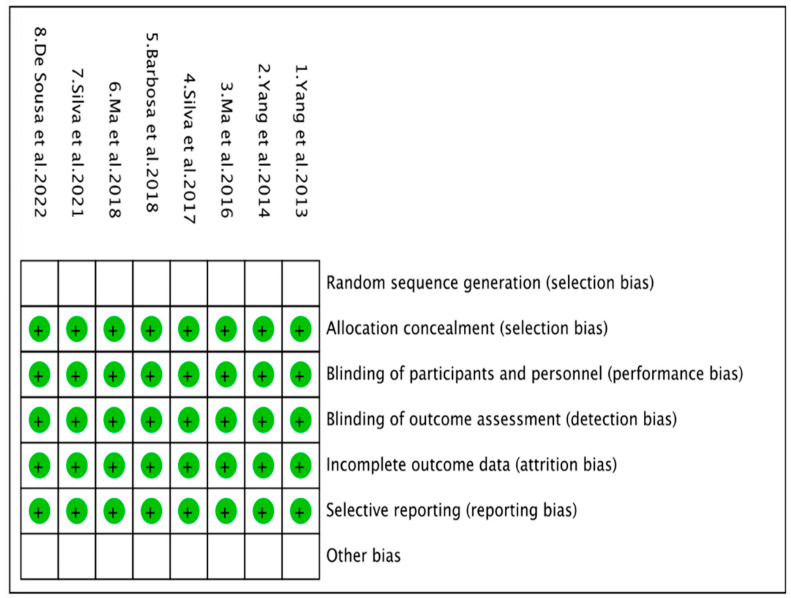
Risk of bias summary: a review of authors’ judgments about each risk of bias item for each included study [5,19,20,21,22,23,24,25].

The recommendations of the Cochrane risk of bias assessment tool were used [20]. Each study was categorized according to the percentage of positive responses to the questions corresponding to the assessment instrument (Figure 2 and Figure 3). Risk of bias was analyzed using RevMan 5.3.0 software developed for Systematic Reviews, available for free download (https://training.cochrane.org/online-learning/core-softwareCochrane%20reviews/revman/revman-5-to%20go%20down, accessed on 24 July 2023). This program was used to detect intervening factors based on the 7 judgment criteria provided by the program, which are: (1) random sequence generation, (2) allocation concealment, (3) subject and staff blinding, (4) blinding evaluation procedures, (5) incomplete results data, (6) selective reporting, and (7) other biases.

## 3. Results

### 3.1. Search Results 

A total of 288 studies were identified between searches in the databases PubMed/Medline (n = 91), Scopus (n = 73), and ScienceDirect (n = 124). After the removal of duplicates (n = 61), 227 articles were screened for the inclusion process. Then, 217 publications were excluded after observing the title/abstract, and the remaining 10 studies were selected for reading the full text. Finally, eight studies were included in the present systematic review. The process of search, selection, and inclusion of studies was summarized in the flow diagram of the PRISMA statement (Figure 1).

### 3.2. Study Characteristics

#### 3.2.1. Characteristics of the Studies Included in Humans

As detailed in Table 2, we observed that the included studies were published between 2014 and 2022. Six studies were carried out in humans, of which, four studies were carried out in Brazil [5,21,22,23] and two in China [24,25]. Four studies utilized cross-sectional methods [5,22,24,25], one study had both cross-sectional and cohort designs [22], and one study used cohort design only [21]. The total number of participants ranged from 22 to 844 subjects, divided according to the different clinical forms of leprosy. In the studies included, there was a heterogeneity of clinical forms of leprosy, including without reaction (WR), type 1 reaction (T1R), indeterminate leprosy (II), tuberculoid leprosy (TT), borderline tuberculoid (BT), borderline lepromatous (BL), lepromatous leprosy (LL), paucibacillary leprosy (PB), and multibacillary leprosy (MB) [5,20,21,22,23,24]. The LL form was the most prevalent, being found in five included studies [5,20,21,22,24]. Five studies used both sexes [5,21,23,24,25]; however, in one included study, sex was not reported [22]. The average age of leprosy patients ranged from 42.9 to 56.8 years old. Three included studies evaluated the bacillary index (BI) and logarithmic bacillary index of skin lesion (LBI). Average BI values ranged from 0 to 4.33. Furthermore, mean LBI values were 0–5.23 among leprosy patients [5,21,23]. Finally, among the six studies, only two presented the treatment status of leprosy patients. One study presented patients only in pretreatment and one study presented patients in pretreatment and on treatment [5,21].

#### 3.2.2. In Vitro Studies

Table 3 shows seven included studies that were performed in vitro [5,19,21,23,24,25,26]. The presented studies were published between 2014 and 2021. Different cell lines were used in the included studies. All seven included studies used isolated PBMC cells [5,19,21,23,24,25,26], two studies used the THP-1 monocytes cells [5,23], one study used isolated CD4^+^ T lymphocyte cells [25], and five studies used isolated *M. leprae* strains [19,21,24,25,26]. We observed a diversity in cell donors in the included studies, although the cell lines were collected from healthy donors in all seven studies [5,19,21,23,24,25,26]. Two studies also included human cells obtained from the American Type Culture Collection [5,23]. And, finally, in three studies, the cells were also donated by patients with different clinical forms of leprosy [5,21,23].

### 3.3. Xenophagy Parameters in Leprosy Patients

#### 3.3.1. Skin Lesion from Leprosy Patients

Table 4 shows a summary of data related to autophagy/xenophagy and leprosy reactions. In studies [5,21,22,23], which analyzed skin lesion samples, tuberculoid patients (TT or T-lep) present an increased expression of autophagy-related proteins Beclin-1, GPSM3, ATG14, APOL1, and TPR, in addition to high LC3-II levels. While in lepromatous patients (LL or L-lep), there is a greater expression of FasL, Caspase-1, 3, and 8, RIP1, and RIP3, MLKL, and BAX, but low levels of LC3-II and high BCL2 expression. In patients who develop a type 1 reaction, the study [21] shows a decrease in LC3 mRNA and several autophagic genes, associated with an increase in TLR2, MLST8, NRLP3, CASP1 and IL1B mRNA, and serum IL-1β. Studies [5,23] indicate an increase in IFN-γ and restored xenophagy and an increase in Il-15 and 13 common autophagic genes, respectively.

#### 3.3.2. *M. leprae*-Stimulated Human Monocytic Cell Line THP-1

Studies [5,23] also analyzed in vitro differentiated macrophages from human monocytic cell line THP-1 (Table 4). While study [5] demonstrated that dead but not viable *M. leprae* induced xenophagy in THP-1 cells, but not in a multiplicity of infection (MOI)-dependent manner, in study [23], high autophagic process-related gene expression (RPTOR, ULK2, ATG16L2, ATG10, ATG7, FKBP15, GPSM1, GPSM2, SEC23B, SQSTM1 and LAMP2) was observed in the presence of IFN-γ, as well as high IL-15 secretion.

#### 3.3.3. Monocyte-Derived Macrophages from Healthy Donors upon Stimulation with *M. leprae*

In Table 4, studies [19,24,25,26] analyzed in vitro cells from healthy donors stimulated with live or heat-killed *M. leprae*. Using *M. leprae* Thai-53 or T-58 strains, as well as strains from T-lep or L-lep patients, it was observed that regardless of the patients’ clinical form, killed *M. leprae* induces xenophagy with the production of proinflammatory IL-1β, IL-6, IL-12, and TNF-α, in addition to high immunity-related GTPaseM (IRGM) and IL-12 expression. On the other hand, live *M. leprae* initially induces xenophagy, primed anti-inflammatory T cell responses via high IL-10 production, in addition to decreasing IRGM, MHC-II, and caspases 3 and 9 expression, which blocked xenophagy and apoptosis.

## 4. Discussion

Leprosy reactions are the most common source of persistent neuropathy, deformity and disability induced by *M. leprae* infection. They are characterized by an inflammatory immune response to degraded components of the bacillus that mostly appear with treatment [27]. Therefore, the identification of biomarkers that can predict the T1R or T2R development in individuals with leprosy is extremely important, contributing to better decisions on treatment strategies and control of irreversible complications [28]. As xenophagy is widely related to the immune response and intracellular pathogen elimination, understanding its relationship with type 1 and type 2 leprosy reactions presents advances in the search for markers for a better prognosis and sequelae prevention.

When multibacillary (MB) patients were followed up for 24 months and classified according to the occurrence or not of reversal reaction (T1R), it was observed that MB patients who developed reversal reactional episodes in the future presented xenophagy blockade and increased inflammasome activation. The xenophagy impairment in the T1R group was associated with an increased expression of the NLR family pyrin domain containing 3 (NLRP3), caspase-1 (p10), and IL-1β production, with IL-1β secretion already being observed at a diagnosis time point, 2–20 months before the reactional episode occurrence [20]. The same results previously observed with another mycobacteria, *Mycobacterium tuberculosis* (MTB), showed that blocking xenophagy inhibited tumor necrosis factor-α (TNF-α) while enhancing IL-1β production in peripheral blood mononuclear cells stimulated with MTB [29]. 

The investigation into the processes involved in autophagy/xenophagy and cell death mechanisms, including apoptosis, necroptosis, and pyroptosis, in the cutaneous lesions of patients with leprosy, and the possible relationship of these mechanisms with leprosy and its clinical progression showed that *M. leprae* can adapt and modulate immune evasion strategies, facilitating its proliferation and reducing immunological surveillance [22]. The results indicated that apoptotic and necrotic marker (FasL, Casp8, RIP1, RIP3, MLKL, BAX, and Casp3) expressions were higher in the lepromatous (LL) than in the tuberculoid (TT) and indeterminate (II) forms. On the other hand, when the xenophagy marker Beclin-1 was analyzed, protein expression was found to be increased in the TT but decreased in the LL form [30]. Suggesting that in severe forms of the disease, the action of cytokines that strongly inhibit macrophage activity, such as IL-10, inhibit the formation of autophagolysosomes, corroborating the results obtained in T1R patients in which impaired xenophagy is directly related to inflammasome activation.

When analyzing the treatment of macrophages with live or killed *M. leprae* stimuli, a difference in xenophagy induction was observed. While killed *M. leprae* preferentially induced proinflammatory cytokines, live *M. leprae* resulted in anti-inflammatory T cell responses, characterized by high IL-10 production, which suppressed xenophagy in a negative feedback loop and allowed the persistence of *M. leprae* [19]. In a second study using live or killed *M. leprae* strains isolated from tuberculoid leprosy (T-lep) and lepromatous leprosy (L-lep) patients, the authors found that live *M. leprae* (regardless of the patient’s clinical form) promotes M2 macrophage differentiation. This skewing was associated with a downregulated IRGM expression, an organizer of the core xenophagy machinery, and reduced autophagosome formation, with lower caspase 3 and caspase 9 activity [26]. Studies showed that IRGM polymorphism was associated with the increased susceptibility to leprosy by affecting inflammatory cytokines, with T-lep patients showing the highest expression, whereas L-lep had the lowest expression of IRGM [24,25]. Moreover, live *M. leprae*-infected macrophages prevented efficient phagocytosis, suppressed inflammation, and inhibited xenophagy and apoptosis [26]. 

Although live *M. leprae* isolated from T-lep or L-lep patients are able to downregulate the autophagic machinery, when analyzing the autophagic mechanisms in these two groups (T-lep and L-lep), large differences are observed that must be much more related to the cytokines produced and M1–M2 macrophage polarization. For example, levels of IFN-γ are significantly raised in paucibacillary T-lep when compared with multibacillary L-lep patients. IFN-γ primes macrophages for inflammatory activation and induces the xenophagy antimicrobial mechanism. LC3-positive autophagosomes and autophagic gene expression were predominantly observed in T-lep when compared with L-lep lesions and skin-derived macrophages. In L-lep skin lesion cells, high expression of *BCL2* (a hindrance to autophagy) was observed together with an inhibition of the autophagic flux. Furthermore, an upregulation of autophagic genes (*TPR*, *GFI1B* and *GNAI3*) as well as LC3 levels was observed in cells of L-lep patients that developed RT1 episodes, an acute inflammatory condition associated with increased IFN-γ levels [5]. This supports previous studies which found that the xenophagy inhibiting IL-10 cytokine is predominant in multibacillary leprosy (MB) compared to high levels of IL-26, IFN-γ, and TNF-α (xenophagy-inducing cytokines) found during paucibacillary tuberculoid leprosy (PB) [31,32].

The ability of activated human macrophages to eliminate intracellular mycobacteria involves the induction of both vitamin D-dependent and -independent antimicrobial responses [33]. Activation of the vitamin D pathway leads to the induction of xenophagy and antimicrobial peptides such as cathelicidin and β-defensin 2, culminating in the bacteria elimination [34]. T-lep lesions are characterized by the expression of antimicrobial genes and the presence of cells undergoing xenophagy [34]. In contrast, in L-lep lesions *M*. *leprae* induces type I interferons and subsequently IL-10, which results in IFN-γ and vitamin D-dependent antimicrobial responses in macrophage inhibition, contributing to bacterial persistence [34]. This corroborates the findings of previous studies that IFN-γ plays a central role in activating the autophagic pathway, especially in tuberculoid leprosy, leading to a more self-limited form.

Despite the fact that a previous study shows that multibacillary (MB) patients who develop T1R during treatment exhibit an xenophagy blockade in skin cells, which results in increased inflammasome activation [21], more recent results from the same research group show that MB patients who progress to T1R had increased levels of IL-15 even before the beginning of the reaction, leading them to hypothesize that IL-15 binds to the IL-15R on CD4+ T cells and contributes to IFN-γ production. Once established, IL-15 production is reduced and IFN-γ acts on host cells by inducing xenophagy [23]. 

Regarding the type 2 reaction (T2R), we were unable to find publications in our search relating to xenophagy and erythema nodosum leprosum (ENL). Study using qPCR to screen a panel of 90 genes related to the immune response in leprosy in RNA-derived peripheral leukocytes of patients with (n = 94) and without leprosy reactions (n = 57) observed that there is a marked signature for type 1 (reversal reactions) in the blood, comprising genes mostly related to the innate immune responses, including type I IFN components, autophagy/xenophagy, and Parkin and Toll like receptors. On the other hand, only Parkin was differentially expressed in the T2R (ENL) group [35].

In order to enhance its persistence within the host and evade the immune response, *M. leprae* promotes the polarization of macrophages toward an M2 phenotype, leading to the inhibition of xenophagy [8]. This phenomenon is particularly characteristic of patients with multibacillary (MB) forms of leprosy, where there is a reduced control over bacillary replication. However, for patients who develop T1R, the data remain uncertain and appear to be more closely associated with the cytokine profile, macrophage polarization, and CD4+ T cell responses than with bacillary death. These findings suggest that xenophagy plays a role in the development of the less severe forms of leprosy and leprosy reactions. Nonetheless, further studies are required to establish a clearer understanding of the relationship between the autophagic process and type 1 leprosy reactions. As for the occurrence of T2R (ENL), our search did not yield any data regarding its association with the autophagic process.

## 5. Conclusions and Future Perspectives

Our search results showed a dichotomy in the development of type 1 leprosy reactions and their relationship with xenophagy, with some data showing that MB patients who developed reversal reactional episodes in the future presented xenophagy blockade and increased inflammasome activation, with IL-1β secretion 2–20 months before the reactional episode occurrence. More recent data, noteworthy from the same research group, show that MB patients who progress to T1R had increased levels of IL-15 even before the reaction began, suggesting that IL-15 binds to the IL-15R on CD4+ T cells and contributes to IFN-γ production and induces xenophagy. As the other data presented suggest a better prognosis in patients where the xenophagy system is activated, would patients with stronger IL-15 production have a better outcome from T1R than those who previously produce IL-1β?

Our study demonstrated that although there are good publications on the type 1 reaction, they remain few and diverse. New studies on the relationship between xenophagy and leprosy reactions are necessary, especially when considering the type 2 reaction (erythema nodosum leprosum).

## Figures and Tables

**Table 1:** Eligibility criteria based on the PICOS strategy.

	Inclusion Criteria	Exclusion Criteria
Population	Humans	Animals and other organisms
Intervention/Exposure	Leprosy	No leprosy
Control	No leprosy patients	-
Outcomes	Autophagy parameters	No autophagy parameters
Study Design	Clinical studies	Reviews; case reports; letters to editors; comments; etc.

**Table 2:** Basic characteristics of participants included in the human studies.

Author, Year [Ref.]	Country	Study Design	Group	n	Clinical Form of Leprosy	Sex (Male/Female)	Age, Mean (Range)	BI, Mean (Range)	LBI, Mean (Range)	Leprosy Treatment Status
Barbosa et al., 2018 [21]	Brazil	Cohort	WR	10	2 BL/8 LL	5/5	42.9 (25–65)	4.19 (1.75–5.85)	4.84 (3.5–5.85)	Pretreatment (24-month follow-up)
			T1R	12	6 BL/6 LL	8/4	44.8 (28–66)	3.67 (1–5.50)	4.68 (3.5–5.95)	Pretreatment (24-month follow-up)
de Souza et al., 2022 [22]	Brazil	Cross-sectional	II	10	10 II	-	-	-	-	-
			TT	10	10 TT	-	-	-	-	-
			LL	10	10 LL	-	-	-	-	-
Silva et al., 2017 [5]	Brazil	Cross-sectional	T-lep	26	26 BT	14/12	51 (20–69)	0 (0–0)	0 (0–0)	26 Pretreatment
			L-lep	28	3 BL/25 LL	22/6	45.71 (21–73)	4.33 (0.50–5.85)	5.23 (2.70–5.90)	28 Pretreatment
			T1R	11	11 BL	7/4	53 (26–70)	1.45 (0–3.75)	2.35 (0–3.80)	2 Pretreatment/9 on treatment
Silva et al., 2021 [23]	Brazil	Cross-sectional and cohort	PB	14	14 BT	6/8	54.5 (8–92)	0 (0–0)	0 (0–0)	-
			MB No Progression	8	4 BL/4 LL	7/1	53.37 (34–65)	2.15 (1.50–5.50)	4.38 (2.85–5.95)	-
			MB Progression	7	4 BL/3 LL	5/2	45.14 (32–69)	2.98 (0.50–4.67)	4.6 (2.7–5.95)	-
			T1R	12	9 BL/3 LL	9/3	49.16 (17–66)	2.66 (0.75–5.85)	2.06 (0–3.8)	-
Yang et al., 2014a [24]	China	Cross-sectional	Healthy Control	432	-	302/163	57.1 ± 7.2	-	-	-
			Leprosy Cases	412	79 PB/333 MB	291/141	56.8 ± 6.8	-	-	-
Yang et al., 2014b [25]	China	Cross- sectional	Healthy Control	46	-	30/16	-	-	-	-
			Leprosy Cases	78	9 TT/25 BT/28 BL/16 LL	52/26	-	-	-	-

BI, bacillary index; LBI, logarithmic bacillary index of skin lesion; WR, without reaction; T1R, type 1 reaction; BL, borderline lepromatous; LL, lepromatous leprosy; II, indeterminate leprosy; TT, tuberculoid leprosy; T-lep, tuberculoid leprosy; L-lep, lepromatous leprosy; BT, borderline tuberculoid; PB, paucibacillary; MB, multibacillary; MB No Progression, MB patients; MB Progression, MB patients diagnosed with T1R during the clinical follow-up.

**Table 3:** Basic characteristics of samples included in in vitro studies.

Author, Year [Ref.]	Sample	Characteristics
Barbosa et al., 2018 [21]	Skin Lesion Macrophages Isolated PBMCs and Monocyte Cultures	MB patients Healthy donors (+ armadillo g-irradiated *M. leprae*)
Ma et al., 2017 [19]	Isolated PBMCs and Monocyte Cultures	Healthy donors (6 males) + live or killed Thai53- strain *M. leprae*
Ma et al., 2018 [26]	Isolated PBMCs and Monocyte Cultures	Healthy donor (1 female) + live or killed *M. leprae* strain from 2 T-lep and 6 L-lep patients
Silva et al., 2017 [5]	Skin Lesion Macrophages Differentiated Macrophages	T-lep, L-lep, and T1R patients Human monocytic cell line THP-1 obtained from the American Type Culture Collection
	Isolated PBMCs and Monocyte Cultures	Healthy donors
Silva et al., 2021 [23]	Skin Biopsies Differentiated Macrophages	PB, MB, and T1R patients Human monocytic cell line THP-1 obtained from the American Type Culture Collection
	Isolated PBMCs and Monocyte Cultures	Healthy donors
Yang et al., 2014a [24]	Isolated PBMCs	Healthy donors + heat-killed T-58-strain *M. leprae*
Yang et al., 2014b [25]	Isolated PBMCs and CD4+ T Cells, Monocytes and Macrophages Cultures	Healthy donors + heat-killed T-58-strain *M. leprae*

MB, multibacillary leprosy; PBMCs, peripheral blood mononuclear cells; T-lep, tuberculoid leprosy; L-lep, lepromatous leprosy; T1R, type 1 reaction; PB, paucibacillary leprosy.

**Table 4:** Xenophagy parameters in leprosy patients.

Author, Year [Ref.]	Leprosy Patients	Cell Type	Autophagy Outcomes
Barbosa et al., 2018 [21]	Multibacillary with reversal reaction (24-month follow-up)	Skin lesion cells and PBMCs	↓ LC3 mRNA and several autophagic process-related genes associated with ↑ *TLR2* and *MLST8*. ↑ *NLRP3*, *CASP1,* and *IL1B* mRNA levels, and ↑ IL-1β serum concentration.
de Sousa et al., 2022 [22]	Indeterminate (II), tuberculoid (TT), and lepromatous (LL)	Skin lesion samples	↑ FasL, caspase-8, RIP1 and RIP3, MLKL, BAX, caspase-3, and caspase-1 in the LL form. ↓ Beclin-1 in the LL and II forms, ↑ in the TT form.
Ma et al., 2017 [19]	In vitro Cell cultures + live or killed *M. leprae* stimuli	Monocytes and T lymphocytes from PBMCS	Killed *M. leprae* infection induced production of proinflammatory IL-1β, IL-6, IL-12 and TNF-α, which ↑ xenophagy. Live *M. leprae* infection also ↑ xenophagy, primed anti-inflammatory T cell responses by ↑ IL-10 which ↓ xenophagy.
Ma et al., 2018 [26]	In vitro Cell cultures + live or killed *M. leprae* strains from Tuberculoid (T-lep) and Lepromatous (L-lep) leprosy patients	Monocytes and T lymphocytes from PBMCs	↑ IRGM and IL-12 expression in macrophages treated by killed *M. leprae* strains, which ↑ xenophagy. ↓ IRGM, MHC-II expression, and caspase-3 and caspase-9 activity in macrophages treated with both live *M.* l*eprae* strains, which ↓ xenophagy and apoptosis.
Silva et al., 2017 [5]	Tuberculoid (T-lep) and lepromatous (L-lep) leprosy patients and type 1 reaction (T1R) patients	Skin lesion cells, human monocytic cell line THP-1, and PBMCs	↑ LC3-II levels via immunofluorescence and BECN1, GPSM3, ATG14, APOL1 e TPR gene expression in T-lep patients. ↑ LC3-I levels in L-lep patients by immunofluorescence, and ↑ BCL2 expression which ↓ xenophagy. ↑ IFN-γ and restored xenophagy levels in L-lep patients who developed the reversal reaction.
Silva et al., 2021 [23]	Paucibacillary (PB), multibacillary (MB) and type 1 reaction (T1R) patients (24-month follow-up)	Skin biopsies, human monocytic cell line THP-1, and PBMCs	↑ autophagic process-related genes: *RPTOR*, *ULK2, ATG16L2*, *ATG10*, *ATG7*, *FKBP15*, *GPSM1*, *GPSM2*, *SEC23B*, *SQSTM1,* and *LAMP2* in *M. leprae*-stimulated THP-1 cells in the presence of IFN-γ, and ↑ IL-15 secretion. ↑ *IL15* mRNA levels in T1R lesions compared to PB and MB groups. Presence of 13 common autophagic genes (*FRS3*, *GFI1B*, *GNAI3*, *GPSM1, GPSM2, LETM2, RASD1, RPTOR*, *SEC23B, SEC24A, TPR, UVRAG,* and *BECN2*) between T1R skin biopsies and stimulated THP-1cells with *M. leprae* and IFN-γ.
Yang et al., 2014a [24]	Paucibacillary and multibacillary	PBMCs	IRGM polymorphism (rs13361189TC and CC genotypes) is associated with ↑ susceptibility to leprosy, and rs13361189CC genotype ↑ leprosy complications. ↑ IFN-γ and IL-4 in *M. leprae*-infected PBMC with rs13361189CC genotype. No differences in the distribution of rs13361189 SNP between paucibacillary and multibacillary forms.
Yang et al., 2014b [25]	Lepromatous lepromatous (LL), borderline lepromatous (BL), borderline tuberculoid (BT), and tuberculoid (TT)	CD4+ T cells, monocytes, and monocyte-derived macrophages from PBMCs	↑ IRGM protein and mRNA levels in monocytes and macrophages upon stimulation with *M. leprae*. ↑ IRGM in monocytes from TT type, then BT type, BL type, and followed by LL type, showing inverse correlation with the severity of the disease.

PBMCs, peripheral blood mononuclear cells; LC3, microtubule-associated protein 1 light chain 3; TLR2, Toll-like receptor 2; MLST8, MTOR-associated protein LST8 homolog; NLRP3, NLR family pyrin domain containing 3; CASP1, caspase 1; IL-, interleukin; FasL, factor-related apoptosis ligand; RIP1 and RIP3, receptor-interacting protein; MLKL, mixed lineage kinase domain-like pseudokinase; BAX, Bcl-2-associated X-protein; TNF-α, tumor necrosis factor alpha; IRGM, immunity-related GTPaseM; MHC-II, major histocompatibility complex class II; BECN1, beclin-1; GPSM, G-protein-signaling modulator; ATG, autophagy-related genes; APOL1, apolipoprotein L1; TPR, translocated promoter region; BCL2, B-cell lymphoma 2; IFN-γ, interferon-gamma; RPTOR, regulatory-associated protein of MTOR complex 1; ULK2, unc-51-like autophagy-activating kinase 2; FKBP15, FKBP prolyl isomerase family member 15; SEC23B, *SEC23* homolog B; SQSTM1, sequestosome 1; LAMP2, lysosome-associated membrane protein; FRS3, fibroblast growth factor receptor substrate 3; GFI1B, growth factor independence 1B; GNAI3, G protein subunit alpha I3; LETM2, leucine zipper and EF-hand containing transmembrane protein 2; RASD1, ras-related dexamethasone-induced 1; UVRAG, UV radiation resistance-associated; BECN2, beclin-2; SNP, single-nucleotide polymorphism.

## Data Availability

All data from the articles used in this systematic review are available in the references.
